# A secreted bacterial protein protects bacteria from cationic antimicrobial peptides by entrapment in phase-separated droplets

**DOI:** 10.1093/pnasnexus/pgae139

**Published:** 2024-04-02

**Authors:** Nicholas K H Ostan, Gregory B Cole, Flora Zhiqi Wang, Sean E Reichheld, Gaelen Moore, Chuxi Pan, Ronghua Yu, Christine Chieh-Lin Lai, Simon Sharpe, Hyun O Lee, Anthony B Schryvers, Trevor F Moraes

**Affiliations:** Department of Biochemistry, University of Toronto, Toronto, ON M5S 1A8, Canada; Department of Biochemistry, University of Toronto, Toronto, ON M5S 1A8, Canada; Department of Biochemistry, University of Toronto, Toronto, ON M5S 1A8, Canada; Molecular Medicine Program, Research Institute, The Hospital for Sick Children, Toronto, ON M5G 0A4, Canada; Department of Biochemistry, University of Toronto, Toronto, ON M5S 1A8, Canada; Department of Biochemistry, University of Toronto, Toronto, ON M5S 1A8, Canada; Department of Microbiology, Immunology and Infectious Diseases, University of Calgary, Calgary, AB T2N 4N1, Canada; Department of Biochemistry, University of Toronto, Toronto, ON M5S 1A8, Canada; Department of Biochemistry, University of Toronto, Toronto, ON M5S 1A8, Canada; Molecular Medicine Program, Research Institute, The Hospital for Sick Children, Toronto, ON M5G 0A4, Canada; Department of Biochemistry, University of Toronto, Toronto, ON M5S 1A8, Canada; Department of Microbiology, Immunology and Infectious Diseases, University of Calgary, Calgary, AB T2N 4N1, Canada; Department of Biochemistry, University of Toronto, Toronto, ON M5S 1A8, Canada

**Keywords:** antimicrobial peptides, phase separation, secreted proteins

## Abstract

Mammalian hosts combat bacterial infections through the production of defensive cationic antimicrobial peptides (CAPs). These immune factors are capable of directly killing bacterial invaders; however, many pathogens have evolved resistance evasion mechanisms such as cell surface modification, CAP sequestration, degradation, or efflux. We have discovered that several pathogenic and commensal proteobacteria, including the urgent human threat *Neisseria gonorrhoeae*, secrete a protein (lactoferrin-binding protein B, LbpB) that contains a low-complexity anionic domain capable of inhibiting the antimicrobial activity of host CAPs. This study focuses on a cattle pathogen, *Moraxella bovis*, that expresses the largest anionic domain of the LbpB homologs. We used an exhaustive biophysical approach employing circular dichroism, biolayer interferometry, cross-linking mass spectrometry, microscopy, size-exclusion chromatography with multi-angle light scattering coupled to small-angle X-ray scattering (SEC–MALS-SAXS), and NMR to understand the mechanisms of LbpB-mediated protection against CAPs. We found that the anionic domain of this LbpB displays an α-helical secondary structure but lacks a rigid tertiary fold. The addition of antimicrobial peptides derived from lactoferrin (i.e. lactoferricin) to the anionic domain of LbpB or full-length LbpB results in the formation of phase-separated droplets of LbpB together with the antimicrobial peptides. The droplets displayed a low rate of diffusion, suggesting that CAPs become trapped inside and are no longer able to kill bacteria. Our data suggest that pathogens, like *M. bovis*, leverage anionic intrinsically disordered domains for the broad recognition and neutralization of antimicrobials via the formation of biomolecular condensates.

**Significance Statement**

In this work, we discovered a new mechanism by which *Moraxella bovis* evades host antimicrobial peptides such as LL37 and lactoferricin. *Moraxella bovis* secretes lactoferrin-binding protein B (LbpB), which sequesters cationic antimicrobial peptides into phase-separated droplets through a highly charged, anionic, intrinsically disordered domain. In this study, we provide evidence of *M. bovis* using liquid–liquid phase separation as a means to protect itself from antimicrobial peptide-mediated cytotoxicity. Protein-based liquid–liquid phase separation is a process by which proteins spontaneously separate into two phases and is a phenomenon more commonly documented in eukaryotes. Homologs of LbpB have been identified in human pathogens such as *Neisseria gonorrhoeae* and *Neisseria meningitidis*, suggesting the potential prevalence of this mechanism of antimicrobial peptide resistance.

## Introduction

Many gram-negative pathogens secrete virulence factors onto their cell surface and into the extracellular space to assist in the colonization of their hosts (1, 2). The Neisseriaceae and Moraxellaceae families secrete a heavily charged surface lipoprotein, lactoferrin-binding protein B (LbpB), that functions in tandem with an outer membrane transporter (LbpA) to acquire iron from host lactoferrin (Lf), an iron sequestering, antimicrobial protein released from neutrophils (3). The LbpB/A bipartate receptor is homologous to the well-studied TbpA/B (transferrin-binding proteins A and B) receptors, but LbpB has also been implicated in other biological roles. In addition to functioning as a nutritional receptor, the negatively charged loops of *Neisseria meningitidis* LbpB have been shown to impart resistance to the cationic antimicrobial peptides (CAPs) derived from host Lf, such as lactoferricin B, which we are denoting Lf(17–41) (4–6). The enzymatic release of *N. meningitidis* LbpB from the cell surface by the bacterial protease NalP is hypothesized to play a role in the ability of LbpB to act as a “CAP sink” (7, 8).

CAPs are a critical component of the innate immune system that function against infection, are typically enriched in hydrophobic and positively charged residues, and are amphipathic, with positively charged residues clustered on a single face (9, 10). The positive character of these peptides is responsible for selectively targeting them to negatively charged bacterial membranes, where the peptides hydrophobic face functions to disrupt the bacterial membrane. In response to this, many common pathogens have adapted to become resistant to their effects through a wide array of resistance mechanisms such as modification of the bacterial surface (11, 12), increased peptide efflux (13, 14), and production of a protective extracellular polysaccharide capsule (15, 16). Bacteria can also intercept CAPs at the extracellular level by degrading CAPs via secreted proteases (17), or sequestering CAPs by direct binding to the components of outer membrane vesicles (18, 19) and biofilms (20, 21), and by binding proteins such as LbpB (8).

The structural studies of LbpBs produced thus far have focused largely on the iron piracy activity of LbpB, and in all cases, either researchers have removed the anionic portions of LbpB for structural elucidation or the anionic portions have not been visible in the final structures (8, 22, 23). This leaves the mechanisms of LbpB-mediated protection against CAPs largely unexplored. In the present study, we show that the anionic regions of LbpB are conserved across a number of pathogenic species and, in the case of *Moraxella bovis* LbpB, protection from CAPs is accomplished by sequestering the peptides into biomolecular condensates. The phenomenon of phase separation in biology is a rapidly advancing field (24), and often involves the formation of discrete compartments called biomolecular condensates (or membraneless organelles) that enrich and exclude certain proteins and nucleic acids. Biomolecular condensates are involved in a myriad of cellular functions (25), have been well documented in eukaryotes, and, more recently, have been described in bacteria (26). The majority of these bacterial examples focus on condensate formation taking place intracellularly; however, there are cases of higher-order eukaryotes of phase separation playing a role outside of the cell (elastin (27), resilin (28), and recently, galactin-3 (29)). Thus, the utilization of phase separation by bacterial pathogens outside the cell to avoid immune factors is intriguing. In this study, we show that the helical and highly charged C-terminal domain in LbpB is partially disordered, which may enable it to sequester CAPs into phase-separated droplets as a powerful strategy for bacteria to escape antimicrobial activity.

## Results

### The anionic region of LbpB is highly conserved across species

The structural layout of several *Neisserial* surface lipoproteins such as LbpB consists of N- and C-terminal lobes, with each lobe being made up of a β-barrel and a β-handle domain (Fig. S1A) (30). For both TbpB and LbpB, the ligand-binding domain is found exclusively on the N-lobe domain (8), while the C-lobe of LbpB is unique in that it contains a highly negatively charged region inserted in the C-lobe. Bioinformatic analysis has shown the presence of one or two anionic regions in the LbpBs of several gram-negative bacteria (4, 31). Studies have demonstrated that the anionic regions of LbpB from *N. meningitidis* and *M. catarrhalis* protected their respective pathogens against CAPs (5, 6). Here, we confirm the presence of highly negatively charged regions in the LbpBs of several species by using a moving average window to calculate the average net charge along the LbpB sequence (Fig. S1B). We find an exception in the cattle pathogen *M. bovis* LbpB, in which the anionic region shows an enrichment in residues that have higher helix-forming propensities. In most cases, the anionic regions of the LbpBs are predicted by PSIPRED and AlphaFold 2.0 to be random coil; however, the corresponding region of *M. bovis* is predicted to have a helical character (32, 33). Of all documented LbpBs, the *M. bovis*-charged region also represents the largest known C-lobe insertion (∼32 kDa), which we name the C-terminal α-helical domain (CaHD).

### The *M. bovis* LbpB protects the bacteria from the antimicrobial activity of CAPs

**Fig. 1.**
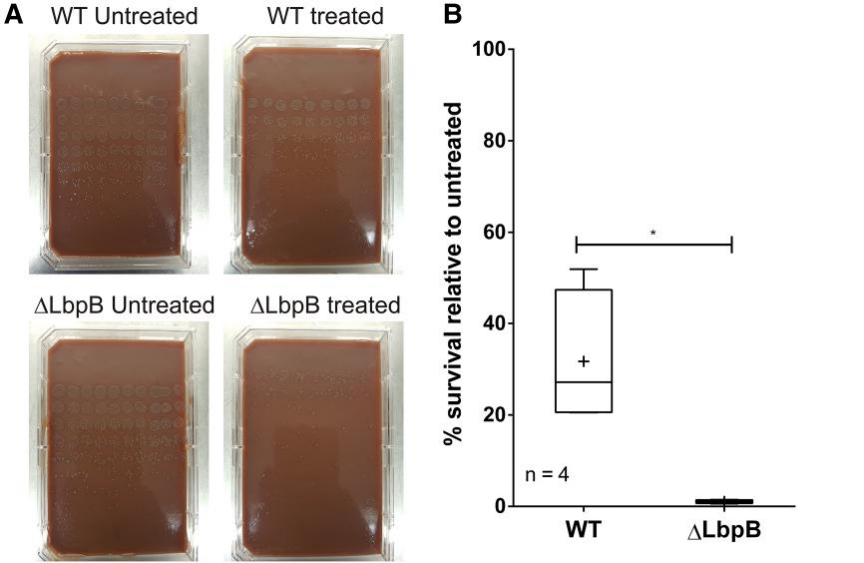
LbpB and CaHD protect bacteria from CAP-mediated killing. A) Killing assay of wild type (WT) and ΔLbpB *M. bovis* treated with lactoferrin hydrolysate, showing that cells lacking LbpB are highly susceptible to killing by proteolyzed lactoferrin. Rows on each plate descending are undiluted, diluted 1/3, 1/9, 1/27, 1/81, and 1/243. B) Quantification of cell survival from four biological replicates.

As the CaHD from *M. bovis* LbpB differed significantly from the anionic regions of previously studied LbpBs, we tested whether the full-length *M. bovis* LbpB also had activity against CAPs (5, 6). Wild-type *M. bovis* and *M. bovis* ΔLbpB were subjected to a killing assay using a concentrated hydrolysate of Lf. Cells were incubated with the killing reagent, and serial dilutions were spotted onto nonselective chocolate plates for colony counting the subsequent day (Fig. 1A). In each replicate treatment, between 20 and 50% of wild-type cells survived and only ∼1% of ΔLbpB cells survived (Fig. 1B), indicating that the *M. bovis* LbpB could also protect bacteria from CAP-mediated cell death.

### CaHD directly binds apo-Lf, holo-Lf, and an Lf-derived CAP

**Fig. 2.**
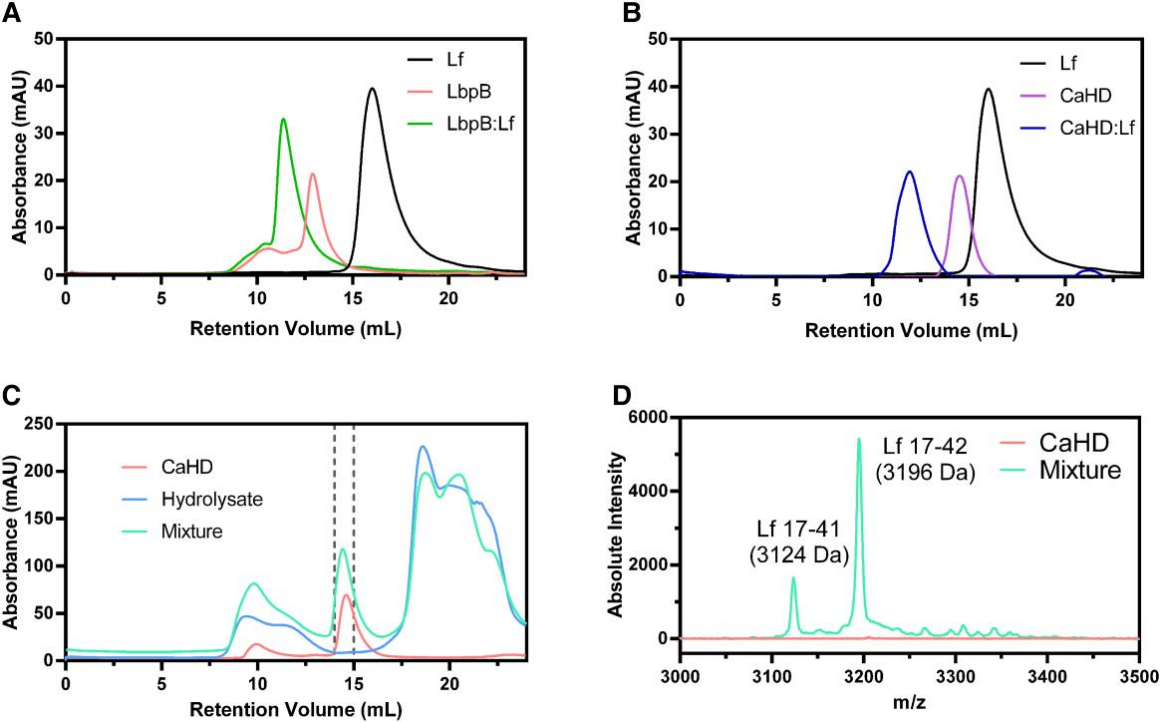
Complex formation of LbpB and CaHD with Lf and Lf(17–41). Size exclusion chromatography of A) LbpB:Lf, B) CaHD:Lf, and C) CaHD:Lf hydrolasate complexes. D) The fractions from 2C, corresponding to the 14–15 mL retention volume from CaHD and the CaHD:Lf hydrolysate mixture were analyzed with MALDI-TOF mass spectrometry.

We hypothesized that LbpB-mediated protection against CAPs was accomplished by sequestering CAPs through direct interactions to the CaHD. A full-length LbpB and CaHD alone were expressed and purified from *Escherichia coli* and examined by size exclusion chromatography. LbpB:Lf (Fig. 2A), CaHD:Lf (Fig. 2B), and CaHD:Lf(17–41) (Fig. 2C) complexes coeluted when passed through a Superdex S200 column. The shift in elution volume of the CaHD:Lf(17–41) complex from CaHD alone was minimal; hence, we confirmed the presence of the Lf(17–41) peptide in the CaHD fraction using mass spectrometry (Fig. 2D).

**Fig. 3.**
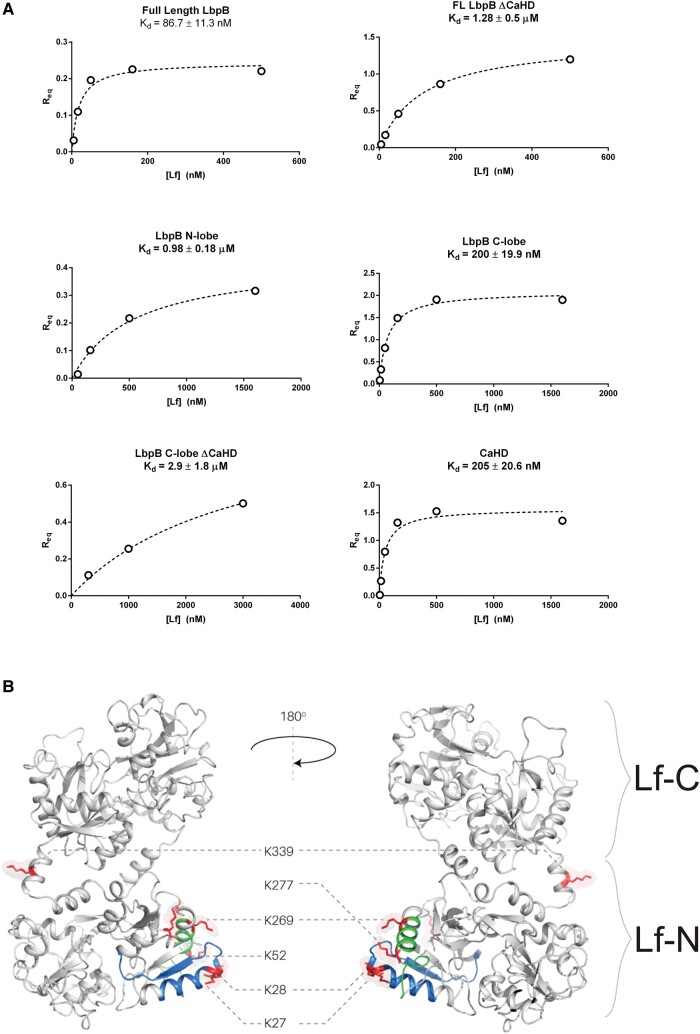
Exploring the binding interface between Lf and *M. bovis* LbpB. A) Steady-state-binding curves between LbpB domains and holo-Lf measured by BLI. B) Lf residues that participated in chemical cross-linking with CaHD. Illustrated lysines that were cross-linked to CaHD primarily cluster around the antimicrobial peptides LfcinB (shown in blue) and Lfampin (shown in green).

Recent structures of *N. meningitidis* and *Neisseria gonorrhoeae* LbpB complexed to human Lf identified that the LbpB N-lobe contained the binding site of Lf(8). To test whether this mode of binding was conserved in *M. bovis* LbpB, we deconstructed LbpB into its constituent domains via subcloning into a 6His-biotin-acceptor peptide (BAP)-MBP vector for use in biolayer interferometry (BLI; Fig. 3A) (34). Each recombinant protein had its affinity for Lf measured outside of the context of the full-length protein. Any recombinant protein containing CaHD had a high affinity (low *K*_D_s) for Lf. Surprisingly, LbpB N-lobe:Lf interactions were roughly 5-fold weaker than that of CaHD:Lf, indicating that the *M. bovis* LbpB may not prioritize iron acquisition through the traditional binding mechanism (35, 36). To further determine the binding interface, the purified LbpB:Lf and CaHD:Lf complexes were disuccinimidyl suberate (DSS)-cross-linked/trypsinized for mass spectrometry analysis. The resultant interprotein cross-links occurred primarily between the Lf N1 subdomain and CaHD (Fig. 3B). Several cross-links mapped directly onto the two antimicrobial peptides, lactoferricin B and lactoferrampin, within the N1 subdomain (37). This was in contrast to the Neisserial LbpB:Lf complexed structures, in which the N-lobe of LbpB bound to the Lf C-lobe (8).

To test whether the presence of iron effected CaHD:Lf binding, a lactoferrin-conjugated sepharose resin was prepared in either the apo or the holo state and incubated with free CaHD. Equivalent amounts of CaHD were pulled down by either resin, suggesting that CaHD shows no preference for iron-loaded lactoferrin (Fig. S2). This is in contrast to the transferrin-binding protein B (TbpB), which binds only the holo form of transferrin (38). Considering the large conformational change that Lf undergoes during the transition from holo to apo (39), this indicates either that CaHD may be binding a region that remains relatively constant in the two states or that the structural dynamics of CaHD allow it to bind Lf in a variety of conformations.

As our binding data implicated CaHD as the main contributor to binding Lf and Lf-derived peptides, we tested whether the presence of CaHD alone could protect cells from CAPs. We added CaHD externally to wild-type K12 *E. coli* in the presence of an Lf(17–41) concentration known to inhibit the growth of the bacteria. The previously calculated minimum inhibitory concentration of Lf(17–41) was found to be 33 µM (40). In our experiment, the growth of *E. coli* cells was inhibited at 50 µM Lf(17–41), but the bacteria was able to grow if CaHD was present in the media (Fig. S3).

### Biophysical and structural studies reveal significant disorder within CaHD

**Fig. 4.**
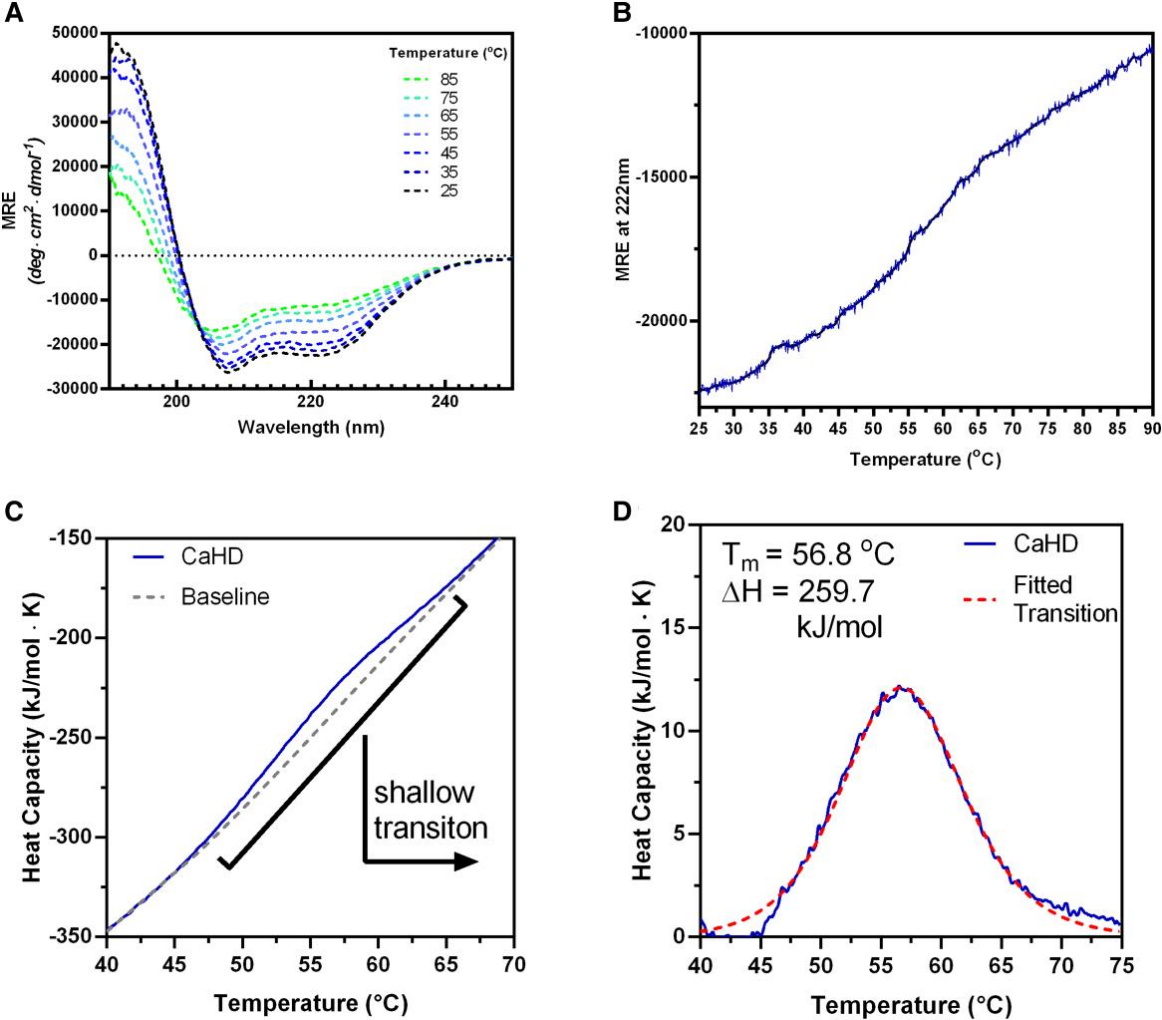
Secondary structure and stability of CaHD. A) Full CD spectra of 0.05 mg/mL of CaHD were collected every 10°C during a temperature melt with a temperature ramp of 1 °C/min. B) The changes in the mean residue ellipticity (MRE) of CaHD at 222 nm as a function of temperature. C) Differential scanning calorimetry of 1 mg/mL purified CaHD (50 mM sodium phosphate, pH 7.4, 200 mM NaCl) using an increasing temperature ramp of 0.5 °C/min. D) A single low-energy transition at 57 °C is observed. Data were fit to a two-state transition.

In order to explore the mechanisms through which CaHD was inhibiting the activity of Lf(17–41), we sought to characterize the domain from a structural and biophysical point of view. Considering the high degree of charged residues in the CaHD, it was unsurprising when our exhaustive crystal screens on full-length *M. bovis* LbpB, CaHD alone, and the CaHD:Lf complex yielded no crystal hits. All experiments failed to yield crystals but did provide indirect evidence of CaHD's highly sampled configuration space and implied that it may be partially disordered. As discussed above, the CaHD of the *M. bovis* LbpB was predicted to have helical content. We verified the predicted helical content of CaHD by circular dichroism (CD). The CD spectra of CaHD reflected that of a helical protein with minima at 208 and 222 nm, and a temperature ramp confirmed that CaHD retained global helical content even at high temperatures (Figs. 4A and B). The melting curve exhibited a linear decrease in helicity, consistent with the gradual unfolding of helices as the temperature increased. A thermal ramp from 10 to 75°C using differential scanning calorimetry showed a single shallow transition at 57 °C (Fig. 4C and D) that was consistent with DSF melts (Fig. S4), suggesting that there may be an unfolding event beyond the unraveling of helices. The enthalpy of this transition was low (259.7 kJ/mol), suggesting that if the helices in CaHD form a globular domain, that only a few nonbonded interactions contribute to its folding resulting in a shallow energy landscape. Apart from the one transition, the heat capacity as a function of temperature increased linearly, consistent with the slow unraveling of helices, similarly observed in the CD melt. Together, these experiments describe a primarily helical domain with a loosely packed globular core.

**Fig. 5.**
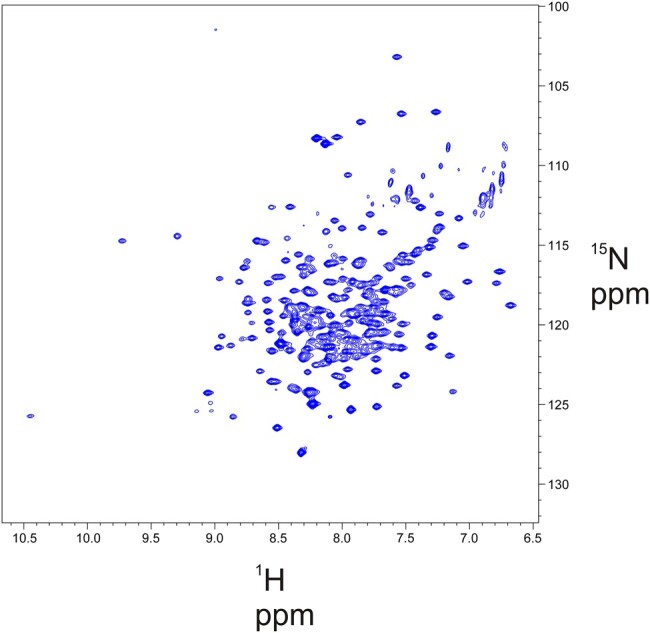
Solution NMR of CaHD. The TROSY ^1^H–^15^N HSQC of 1 mM CaHD in buffer containing 50 mM sodium phosphate buffer (pH = 7.0), 50 mM NaCl, 50 mM arginine, 50 mM glutamate. The shows a mix of well dispersed peaks and overlapping peaks clustered between 7.5 and 8.5 ppm in the ^1^H dimension.

We next performed solution NMR as an alternative approach to structurally resolve the nature of the CaHD. Recombinant CaHD was uniformly labeled with ^15^N and ^13^C isotopes for characterization by NMR spectroscopy. Backbone resonance assignment was completed using standard triple resonance NMR experiments. Assigned chemical shifts were used to calculate secondary chemical shifts along the length of CaHD and showed a mixture of helices and disordered regions (Fig. S5). The TROSY ^1^H–^15^N HSQC (Fig. 5) does show a characteristic lack of chemical shift dispersion in the proton dimension that is commonly found in intrinsically disordered proteins (41) with many of the ^1^H shifts clustered between 7.5 and 8.5 ppm. Together, the NMR and CD data demonstrate that CaHD is a collection of helices. However, the NMR data also suggest that portions of the CaHD are disordered.

**Fig. 6.**
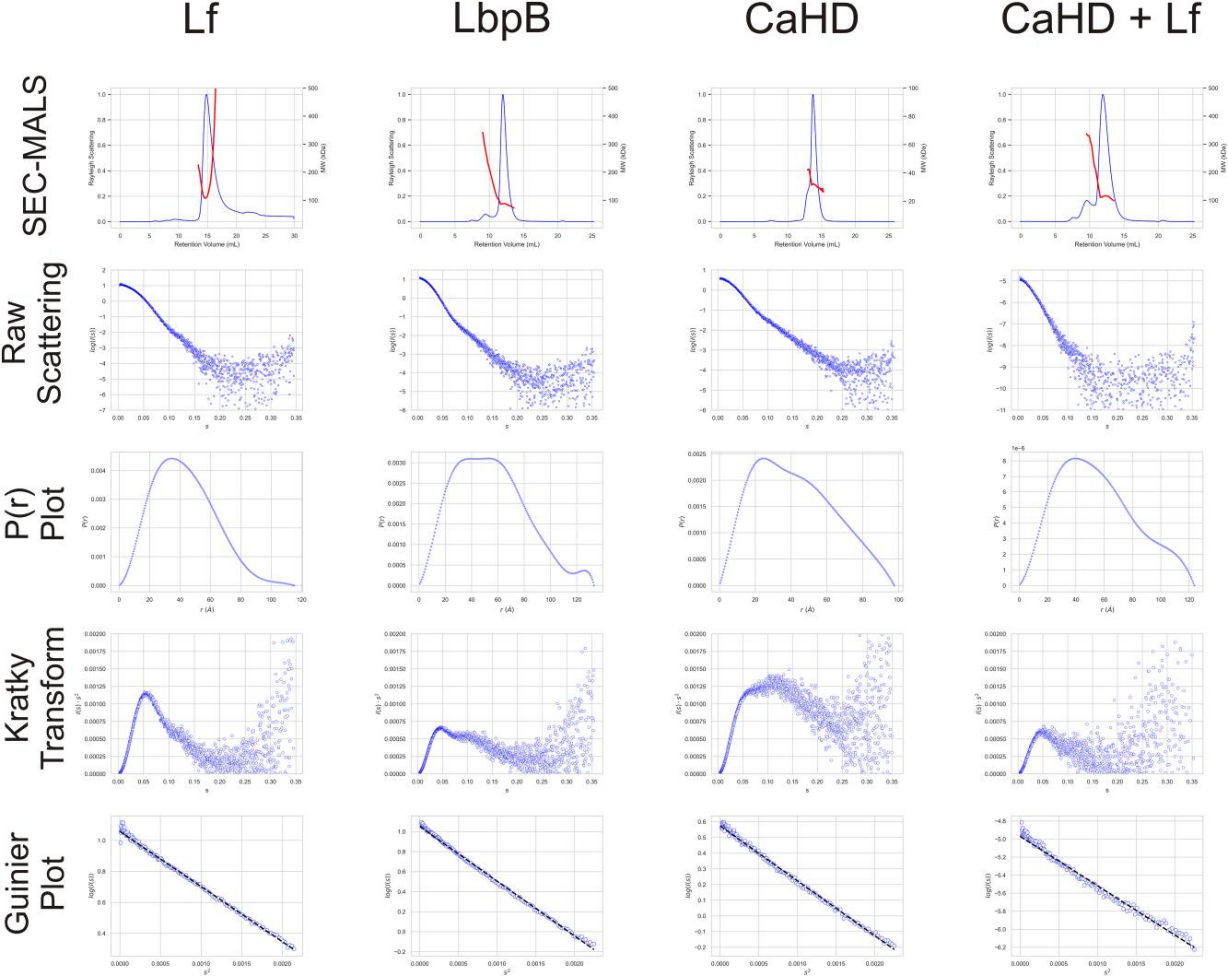
Small-angle X-ray scattering analysis of CaHD, full-length LbpB, Lf, and the CaHD:Lf complex. From top to bottom, plots are as follows: SEC–MALS on a GE S200 increase 10/300, with molecular weight estimates shown above scattering peaks; raw SAXS scattering (log(I(s))); pairwise electron distribution function *p*(*r*); Kratky plot; Guinier plot. Raw scattering is the log-transformed intensity values from the detector. *p*(*r*) represents the fraction of electrons that lie within a distance *r* from another electron. The Kratky plot gives a qualitative measure of flexibility, with globular proteins exhibiting well-defined Gaussians and flexible proteins plateauing at higher scattering angles. The Guinier plot represents the low *q*-regions of scattering (smallest angles) and is used to estimate Rgyr as well as assess aggregation and particle repulsion.

Small-angle X-ray scattering is a powerful tool for the analysis of flexible systems including intrinsically disordered proteins. Purified LbpB, CaHD, Lf, LbpB:Lf, and CaHD:Lf complexes were measured using size-exclusion chromatography with multi-angle light scattering coupled to small-angle X-ray scattering (SEC–MALS-SAXS; Fig. 6). SEC was conducted to remove aggregates and prepare oligomeric states for MALS measurements, which revealed information on compositional monodispersity and molecular weight/stoichiometry, and SAXS in order to obtain structural information. Measurements of Lf, LbpB, and CaHD in isolation were consistent with a monomeric state (Fig. 6). In all cases, except Lf alone, the molecular weight measurements were relatively constant across the peak. The large positive charge carried by Lf at physiological pH makes it highly prone to nonspecific interactions which we circumvented by blocking with dilute glutaraldehyde. This was not the case with the CaHD:Lf or LbpB:Lf complexes, as we suspect that the positively charged surface is occluded in the complex, consistent with cross-linking data. The CaHD:Lf complex had an MW of 110 kDa and thus 1:1 stoichiometry. The raw scattering data for each measurement was transformed to create a Guinier plot, Kratky plot, and pairwise electron distribution function, *p*(*r*). All Guinier plots showed no signs of significant aggregation nor particle repulsion. Kratky plots showed the following: Lf—entirely folded, LbpB, CaHD, and CaHD:Lf—a mix of folded and unfolded. The Kratky plots are consistent with a model in which the N-lobe of LbpB is well-folded, but the C-lobe, although helical, is highly flexible and conformationally heterogeneous.

*Moraxella bovis* LbpB CaHD uses multiple mechanisms of protection against Lf and Lf-derived CAPs.

**Fig. 7.**
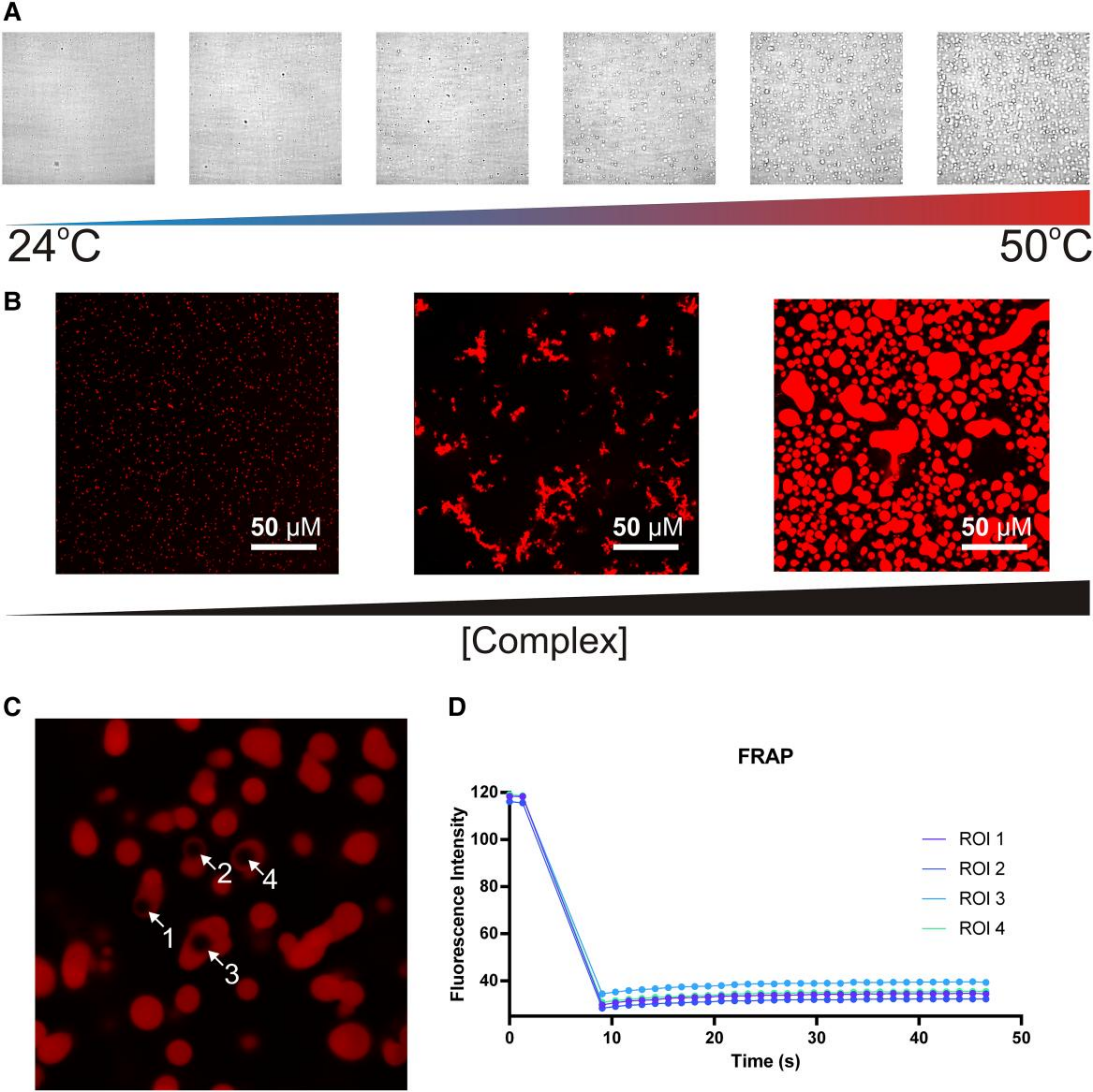
Observing the phase separation of CaHD and Lf(17–41) complexes. A) Brightfield microscopy of a 150-µM CaHD, 180 µM Lf(17–41) mixture at increasing temperatures (24 to 50°C). B) Fluorescence microscopy of fluorophore-labeled CaHD coacervating with Lf(17–41), showing localization of CaHD within the condensates. The number and size of droplets increase with increasing concentrations of CaHD:Lf complex. C) Four regions of interest (ROIs) were photobleached and allowed to recover. D) The quantification of the FRAP measurements collected at the ROI indicated in **4C** showing almost no diffusion of CaHD back into the photobleached region.

- *The CaHD:Lf interaction inhibits the release of CAPs from Lf by shielding Lf from proteases*As CaHD was shown to bind full-length Lf as well as Lf(17–41), we hypothesized that CaHD could serve as a “shield” over the Lf N1 subdomain of intact Lf and could occlude proteases from liberating CAPs from Lf. Pancreatic elastase was used to digest Lf in the presence and absence of CaHD or an unrelated proteinaceous substrate for 24 h. We then assessed the total remaining intact Lf by sodium dodecyl sulfate–polyacrylamide gel electrophoresis (SDS–PAGE) and checked for Lf(17–41) using matrix assisted laser desorption ionization mass spectrometry (MALDI-TOF MS). We found that the presence of CaHD decreased the overall digestion of intact lactoferrin relative to the control and inhibited generation of CAPs (Fig. S6). Although this mechanism of protection is unlikely to be relevant to our in vitro killing assays, it may play a significant role in the host organism.
- *CaHD cocondenses with CAPs into gel-like condensates*
  Intrinsically, disordered regions have been shown to promote phase separation by providing multivalent interaction surfaces, although not all disordered regions promote phase separation (42). We did not find any evidence that CaHD was able to undergo phase separation in isolation but when attempting to characterize the interaction between CaHD and purified peptide, (Lf(17–41)), we found that mixing the protein with peptide resulted in a hazy solution. Initially, this was suspected to be the result of aggregation. However, we found that by altering the temperature, the turbidity could be increase/reduced and that this phenomenon was indefinitely reversible. These results are not consistent with what is expected of aggregation and prompted us to examine this phenomenon further. When CaHD and commercially synthesized Lf(17–41) were mixed and observed under brightfield microscopy, we observed the presence of spherical condensates (Fig. 7A). With increasing temperature, the size of the droplets quickly increased and began to settle to the bottom of the cover slip. Altering temperature allowed us to reversibly change sample turbidity and droplet formation.

We sought to capture portions of the phase space landscape by measuring turbidity as a function of temperature (24 to 50°C), CAP concentration (50 to 300 µM), CaHD concentration (7 to 150 µM), and NaCl concentration (80 to 180 mM) (Fig. S7). We found that the coacervation process was sensitive to the aforementioned conditions and occurred within physiological boundaries (37°C and 150 mM NaCl). We discovered that any of the protein or peptide participants in isolation did not undergo phase separation and that full-length Lf mixed with CaHD also did not result in phase separation (Fig. S7A–E). When full-length LbpB was mixed with Lf(17–41), phase separation was observed, increasing the concentration of peptide resulted in an increase in phase separation/turbidity (Fig. S7F). As both protein and peptide components were required to observe phase separation, we can deduce that the phenomenon is some type of complex coacervation. To determine whether this complex coacervation was unique to Lf(17–41) or whether it could be observed using other CAPs, we repeated this experiment using full-length LbpB and LL37, a well-studied mammalian CAP that has no sequence homology with Lf(17–41). We saw the same effect as with LbpB/Lf(17–41), increasing peptide concentrations resulted in increased amounts of phase separation (Fig. S7G and H).

The presence of both anionic protein (LbpB/CaHD) and cationic peptide (LL37/Lf(17–41)) was required in order to observe phase separation. Increasing peptide concentration in a fixed amount of protein (LbpB or CaHD) (Fig. S7F–H) or increasing the total amount of protein:peptide complex (Fig. S7I) resulted in an increase in the amount of phase separation observed. Strangely, increasing the amount of CaHD in a constant amount of Lf(17–41) results in a loss of turbidity at lower temperatures, but turbidity returns as the temperature is increased (Fig. S7K and L). In situations where there are high concentrations of peptide relative to CaHD, phase separation is favored (Fig. S7K and L), suggesting that multivalency is driving-phase separation. To elaborate further, it may be a requirement that multiple peptides bind to CaHD in order to drive phase separation; however, the mechanism for this remains unclear. One possibility is that the presence of a peptide neutralizes the charges present on CaHD, allowing hydrophobic interactions to form. This hypothesis is supported by the fact that higher salt concentrations are inhibitory to phase separation (Fig. S7J).

To understand the dynamics within the coacervates, we added a cysteine residue to the C-terminus of our CaHD construct for maleimide-based fluorescent labeling. The diffusive kinetics within the condensate were measured using fluorescence recovery after photobleaching (FRAP). After performing a laser pulse on four regions of interest (ROIs), the ROIs’ fluorescence did not recover to the initial value over the measured time (Fig. 7C and D), “FRAP” panel, indicating that the internal dynamics within the condensate are extremely slow. This suggests that the condensates may serve in a “sequestration” role, consolidating CAPs into an extracellular compartment that limits back-exchange into the external milieu.

## Discussion

We discovered a previously uncharacterized domain within *M. bovis* LbpB (CaHD) that exhibited low sequence complexity, enrichment in disorder-promoting/acidic residues, high solubility, conformational heterogeneity, multiple binding partners, and a propensity to undergo complex coacervation via polyelectrostatic interactions (42–45). CaHD is not entirely disordered nor entirely structured—it is perhaps this property that enables CaHD to promiscuously form complexes with apo-Lf, holo-Lf, Lf(17–41), and LL37—each of which are structurally distinct. *Moraxella bovis* benefits from secreting a polypeptide chain of amphipathic “sticky helices” as it may enable the bacteria to produce a single protein capable of recognizing a wide range of substrates (Lf and CAPs), including those not studied here. This promiscuous binding allows for adaptive recognition of CAPs and potentially broadens the niches in which the bacterium can inhabit. This method of defense is arguably more efficient than genetically storing and evolving separate high-affinity cognate proteins for each host CAP—thereby risking loss of immune escape via mutation by either organism.

**Fig. 8.**
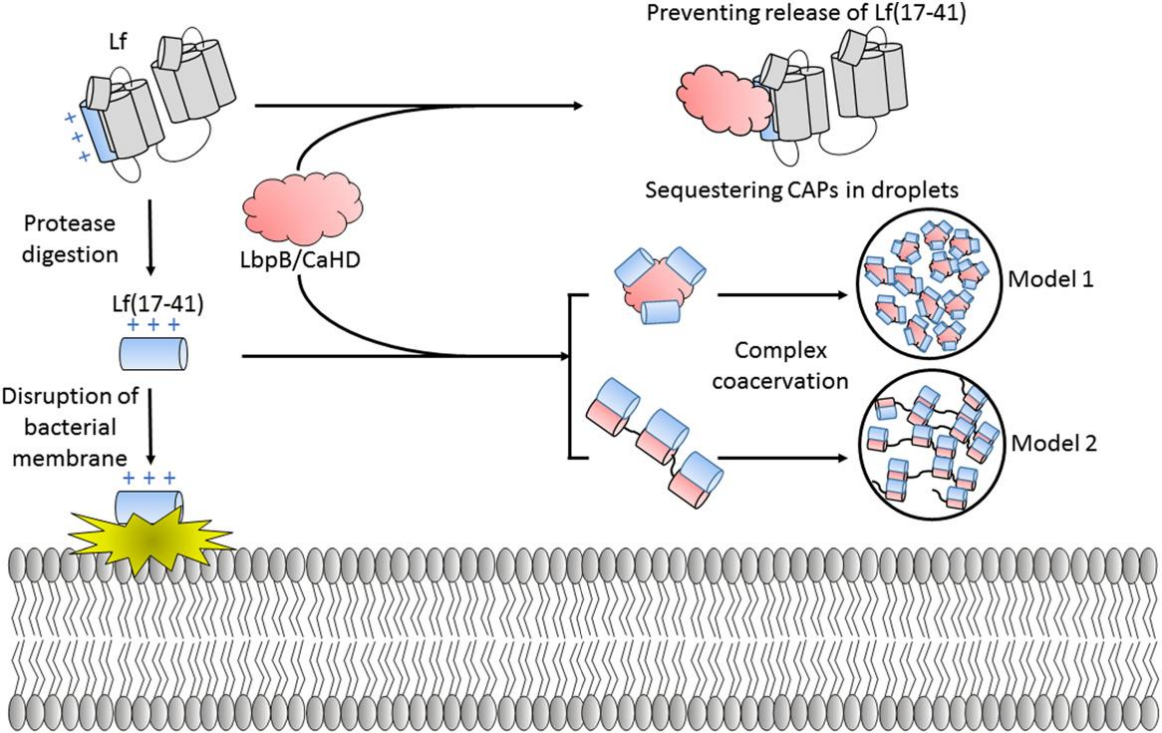
Mechanisms of LbpB-mediated protection from CAPs. Our data support a model in which LbpB/CaHD can protect bacteria from Lf-dervied CAPs through multiple mechanisms. Firstly, LbpB/CaHD can bind to full-length Lf and prevent the proteolytic release of Lf(17–41) (top row). Additionally, if Lf(17–41) and potentially other CAPs (i.e. LL37) are released, LbpB/CaHD can sequester the CAPs in phase-separated droplets (bottom rows). Multivalency in this system may be achieved in a model in which the helices of CaHD are in a globular bundle, and CAP-binding shields repulsive charges and allows coacervation to occur (model 1). Alternatively, if the helices of CaHD may become extended upon CAP binding and allow for multivalent interactions to form (model 2).

We observed the formation of coacervates under physiologic conditions when LbpB was mixed with Lf(17–41) and LL37. The size and morphology of the coacervates were found to be dependent on the concentration of each partner (and thus stoichiometry), temperature, and salt concentration. Coacervates are known to play key roles in various essential extracellular and intracellular biological processes in organisms ranging from humans to marine life (25). These gel-like condensates may serve as nonspecific CAP sinks, considering biomolecules can be accumulated within condensates at concentrations that are orders of magnitude higher than in the external milieu (46). Additionally, the slow diffusive kinetics within the condensate suggest that the condensates may inactivate the CAP by “trapping” them. Both CaHD and Lf(17–41) are highly oppositely charged polymers, and the amphipathic nature of each of them would allow for multivalent interactions. From our current data, it is unclear how multivalent interactions are forming, and the structural data (CD/NMR/SAXS) are consistent with a model in which CaHD is made up of helices spaced in between disordered regions. It is unclear whether the helices form a tight bundle that can aggregate into coacervates when CAPs are bound, or whether the helices are in an extended state that would facilitate multivalent interactions with other CaHD:peptide complexes (Fig. 8).

With the knowledge that LbpB from *N. meningitidis* is purposefully released from the membrane by the bacterium (via NalP (7)), there is likely a selective advantage for mechanisms that encourage delocalization of LbpB from the membrane. It is not known whether *M. bovis* harbors its own version of NalP, but the highly disordered linker regions connecting LbpB to the membrane and CaHD to the C-lobe could be proteolyzed by either bacterial or host proteases—releasing LbpB and/or CaHD into the milieu.

Given the shared homology with TbpB, we expected the LbpB N-lobe to bind Lf. Our BLI results do demonstrate binding between the *M. bovis* LbpB N-lobe and bovine Lf, which is in agreement with recent *N. meningitidis* LbpB:human Lf complex structures (8); however, the binding is tighter to the C-lobe. It is not clear whether *M. bovis* can extract iron from Lf bound to the LbpB C-lobe, and coupled with the observation that CaHD can bind strongly to apo- or holo-Lf suggests that *M. bovis* is favoring the antimicrobial activity of LbpB over its ability to scavenge iron from the host. The CaHD region is present in the LbpBs of *M. bovis*, *Moraxella bovoculi*, *Moraxella caprae*, and other bacterial pathogens that cause ocular disease in livestock. This is especially interesting because cattle produce almost no lysozyme in the tear film, which places a primary dependence on Lf for antimicrobial action (47). The observation that *M. bovis* readily sequesters Lf and its derivative peptides helps to explain why disease progresses so readily in these cattle.

The previously held notion that bacteria could not evolve resistance to CAPs, since CAPs lack specificity to single protein targets (48), has largely been dismissed (49–51). Consistent with other examples of CAP-resistance mechanisms, LbpB appears to have evolved to target the fundamental requirements of CAPs: their intrinsic positive charge and hydrophobicity. The production of a single protein that broadly protects against CAPs could also be a resistance mechanism that could be exchanged through horizontal gene transfer, although the recipient bacteria would need to already have the secretion machinery (Slam) to place the LbpB in the correct extracellular location to be most effective.

Recent advancements (reviewed in Ref. (52)) provide evidence that the activity of CAPs is more specific than previously thought, and that there is a larger degree of host/pathogen adaptation occurring than previously believed. Despite the apparent broad function of LbpB against CAPs, there are noticeable differences across the C-lobes of LbpBs from related species (Fig. S1B). Whereas *M. bovis* (and others) appear to have one large anionic loop, *N. meningitidis* and *N. gonorrhoeae* have two smaller unstructured anionic loops. Previous studies have shown that the presence of either of the loops is sufficient to impart activity against CAPs (5) and raises the question of whether or not the differences between LbpBs are an adaptive response by the bacteria to the CAP arsenal employed by their respective hosts. Nevertheless, the unstructured/anionic nature of the LbpB C-lobe is conserved across many species, and we believe these other LbpBs will also have activity against CAPs, although we hesitate to predict whether the mechanism of protection (i.e. peptide sequestration into phase-separated droplets) will also be conserved. In the future, it will be critical to explore whether the phase separation mechanisms used by the *M. bovis* LbpB extend to the LbpBs from other species, such as the human pathogens *N. meningitidis* and *N. gonorrhoeae*. Finally, it will need to be tested whether the breadth of LbpB-mediated protection extends to other positively charged antimicrobials, such as other classes of CAPs (i.e. defensins), biocides, or antibiotics.

## Materials and methods

### *Moraxella bovis* killing assay

*Moraxella bovis* strains N111 (wild type) and N320 (ΔLbpA/B) were streaked fresh on a chocolate plate from a glycerol stock kept at −80°C, and grown for 18 h at 37°C and 5% CO_2_. A small inoculum from this plate was transferred into 5 mL of liquid brain heart infusion (BHI) media supplemented with 200 µM of the iron chelator 2,2-bipyridine and 1% IsovitaleX (BBL; 211876), and grown again in a capped and parafilm-sealed 50 mL conical tube for 18 h until the OD_600_ reached 2.0. Cells from liquid culture were diluted to an OD_600_ within the linear range of the spectrometer and subsequently diluted again to obtain an OD_600_ of 0.2. Subsequently, 100 µL of cells were placed in 1.5 mL Eppendorf tubes, and the killing reagent was prepared by dissolving 40 mg of lyophilized Lf peptic digest in 1 mL of sterile MilliQ and filtering it through a 0.22-nm filter into a glass vial to prevent hydrophobic cationic peptides from binding to plastic. One hundred microliters of cells were incubated with 100 µL of the killing reagent for 45 min at 37 °C in capped 1.5 mL Eppendorf tubes with agitation every 15 min. One-third serial dilutions were spotted onto nonselective chocolate plates for colony counting the subsequent day.

### Cloning and expression of LbpB and CaHD

Regions of the *lbpB* gene from *M. bovis* strain N114 genomic DNA were PCR-amplified and cloned into (i) pET52-b(+) (10His); (ii) a custom expression vector (10His-MBP-TEV); and (iii) a custom expression vector (10His-BAP-MBP-TEV), where BAP is a biotin-acceptor peptide that gets biotinylated in vivo. The recombinant plasmids were used to transform *E. coli* strain BL21 (LbpB) or C43 (CaHD) and after a 1-h incubation in LB broth, 200 µL was directly inoculated into a 60-mL starter culture of LB. After growth at 37 °C for 18 h, the cells were re-inoculated into a 1-L culture of ZY auto-induction media in a 1:100 ratio and grown for 24 h. Cells were spun down at 11,000×RCF, the broth supernatant was decanted, and cell pellets were re-suspended in 50 mM sodium phosphate, 300 mM NaCl, 10 mM imidazole, pH 7.4 buffer (resuspension buffer), and lysed using an Avestin Emulsiflex-C3 homogenizer. Lysates were spun at 48,200 × RCF for 1 h, and the supernatant was filtered through a 0.45-μm filter. Filtered sample was loaded onto a nickel resin column from GE using a peristaltic pump or onto free resin in a gravity column. Resin was washed in resuspension buffer and wash buffer (50 mM sodium phosphate, 300 mM NaCl, 30 mM imidazole, pH 7.4) until UV signal baselined and eluted with elution buffer (50 mM sodium phosphate, 300 mM NaCl, 250 mM imidazole, pH 7.4). The eluted protein solution was dialyzed into 20 mM HEPES (4-(2-hydroxyethyl)-1-piperazineethanesulfonic acid) pH 7.4, 100 mM NaCl.

### Complex formation of LbpB/CaHD with Lf/Lf (17–41)

Purified protein samples were mixed in a 1:1 molar ratio and injected onto a Cytiva Superdex 200 10/300 column in a running buffer of 20 mM HEPES pH 7.4, 150 mM NaCl. The hydrolysate for the CaHD:peptide sample was generated in a manner similar to the hydrolysate in the killing assay and mixed with CaHD with the hydrolysate in excess. One microliter of solution from the fractionated peak was mixed with 1 μL of α-cyano-4-hydroxycinnaminic acid on a stainless-steel target plate and dried for MALDI-TOF analysis.

### Biolayer interferometry

BLI experiments were performed on a ForteBio Octet RED 96 machine (Pall Biosciences). C43 cells were transformed with IPTG (isopropyl ß-D-1-thiogalactopyranoside)-inducible plasmids encoding the various recombinant proteins, including a BirA BAP, and plated on LB-Agar plates. A set of five colonies was selected from each plate and inoculated into 5 mL of auto-induction media. Cells were grown for 18 h at 37 °C. Bacterial pellets were resuspended in lysis buffer [1× PBS (phosphate buffered saline), 1% Triton X-100]. Lysozyme, DNase, and PMSF (phenylmethylsulfonyl fluoride) were added to aid in lysis, decrease viscosity, and prevent proteolysis. Resuspensions were incubated at room temperature for 15 min, and spun down in a tabletop centrifuge at 16,100 × RCF for 25 min. The concentrations of Lf for the dilution series were chosen as to flank the *K*_D_ value previously observed for TbpB:hTf and LbpB:hLf interactions (50 nM) by ∼10-fold in each direction. Concentration values were calculated on a logarithmic scale incrementing by 0.5 to obtain the values 5, 16, 50, 160, and 500 nM. Constructs, which exhibited weaker binding, had affinities tested using an analyte concentration 10-fold higher. After an initial baseline in 1× kinetics buffer [1× PBS pH 7.4, 0.002% Tween-20, 0.1 mg/mL BSA (bovine serum albumin)] and sensor loading, assay steps followed a baseline; association; dissociation; regeneration; repeat pattern where regeneration was carried out in a 100-mM sodium citrate pH 3.0, 50 mM EDTA (ethylenediaminetetraacetic acid) buffer, 500 mM NaCl. Association and dissociation steps were carried out in kinetics buffer. Steady-state values were obtained by averaging the response values secured in the last 5 s of the association step and were plotted against the concentration to generate saturation binding curves, and the data were fitted using GraphPad Prism. The “one-site-specific-binding” saturation curve was used to fit each set of binding data after subtraction of nonspecific binding from a negative control run. *Actinobacillus pleuropneumoniae* TbpB was used to nonspecifically bind Lf, and the signal from this binding was subtracted from all other real-time curves. This was necessary because cationic regions on Lf make it prone to participate in nonspecific interactions.

### Protein cross-linking and liquid chromatography with tandem mass spectrometry

The LbpB:bLf and CaHD:bLf complexes were purified in an amine-free buffer (50 mM sodium phosphate pH 7.4, 50 mM NaCl). A stock of DSS (Thermo) at 25 mM in DMSO was prepared and added to a final concentration of 1 mM. This mixture was incubated without shaking for 30 min at 24°C after gentle mixing. The DSS was then quenched using a 1-M NH_4_HCO_3_ solution added to a final concentration of 50 mM. Samples were run on a 4% stacking/10% resolving SDS–PAGE gel. The gel was stained with Coomassie blue, and the band of interest was excised with a clean scalpel blade for processing by conventional tryptic in-gel digestion methods at the Southern Alberta Mass Spectrometry Center, followed by analysis using an Orbitrap Velos mass spectrometer, equipped with an EasyLC1000 nano-chromatography system (Thermo Scientific). Briefly, digests were reconstituted in 0.1% formic acid and loaded on an 8 cm × 75 mm self-packed picotip column (Aeris Peptide XB-C18, 3.6 mm particle size, Phenomenex). Separation was achieved using a 30-min 5–60% gradient of mobile phase B (97% acetonitrile with 0.1% formic acid) at 300 nL/min. Mobile phase A consisted of 3% acetonitrile and 0.1% formic acid. The mass spectrometer was operated in positive ion mode, with a high/high configuration, where MS resolution set at 60,000 (400–2,000 *m*/*z*) and MS2 resolution at 7,500. Up to 10 of the most abundant ions were selected for fragmentation using higher energy collisional dissociation, rejecting charge states 1 and 2, and using a normalized collision energy of 40%. Data analysis was performed using the cross-linking plugin within Mass Spec Studio 2.0, using default settings. Each cross-linked peptide pair was manually inspected for data quality and correct assignment of linked residues, using the manual validation user interface in the studio. The final cross-linked peptides as well as each unique pair of cross-linked residues were exported in .CSV format for use in downstream analysis.

### Affinity capture

Lf-conjugated resins were generated using cyanogen bromide activated Sepharose 4B resin (GE Healthcare), according to the manufacturer’s directions. Iron-free (apo) resins were generated by washing iron-loaded resin in a low pH buffer (1% sodium citrate, 100 mM EDTA, pH 3.0). One hundred milliliters of 50% slurry were washed three times with binding buffer (10 mM sodium phosphate, 100 mM NaCl, pH 6.0) by spinning the resin at 6,000×RCF for 1 min, decanting the supernatant, and resuspending resin in binding buffer. Approximately 100 µg of free CaHD protein was incubated with the resin for 2 h at 4°C. Samples were spun and the supernatant was decanted. Resins were then washed three times in binding buffer with salt adjusted to 120 mM for 20 min. Eighty-five milliliters of 2% SDS-loading buffer (100 mM Tris-HCl, 4% SDS, 0.2% bromophenol blue, 20% glycerol) were added to the resin, and boiled for 5 min to release bound protein.

### LLPS optical microscopy

Brightfield images of coacervates were acquired on an inverted Nikon Eclipse Ti-2A spinning disk confocal microscope with an Andor Zyla 4.2 sCMOS camera using an oil immersion objective (Nikon, NA of 1.40). Ten milliliters of sample (150 µM CaHD, 180 µM Lf(17–41)) were placed onto 35-mm glass-bottom dishes (MatTek) and sealed with an 18-mm No. 1 microcover glass (VWR). Samples were imaged in a Bold Line stage top incubator and objective heater (Okolab) that was pre-equilibrated to 25°C, then increased to 50°C while imaging. FRAP was conducted at 24°C, pulsing three times with 50% laser intensity and then collecting 30 intensity measurements over the course of 38 s.

### Small angle X-ray scattering

Individual proteins and complexes were purified and flash-frozen in liquid nitrogen prior to being shipped to the Advanced Photon Source beamline 18ID (Lemont, IL, USA). Samples were thawed and centrifuged immediately before injection onto a SEC–MALS–DLS–RI–SAXS instrument using a Cytiva Superdex 200 10/300 column in a running buffer of 20 mM HEPES pH 7.4, 150 mM NaCl. MALS–DLS–RI data were measured by DAWN HELEOS-II and Optilab TrEX instruments. Scattering within the *q*-range of 0.0043 to 0.35 was collected on a Pilatus3 1 M (Dectris) detector with a wavelength of 1.033 Å. Radial averaging and buffer subtraction were performed with BioXTAS RAW1.6.3. Guinier and Kratky plots were generated in custom software written in Python.

### Turbidimetry

CaHD at a final concentration of 7.5 to 150 µM was mixed with Lf(17–41) at various concentrations (between 0 and 500 µM in a 20-mM HEPES pH 7.4, 100 mM NaCl buffer). The samples were placed in a transparent flatbottom 96-well plate for optical density measurements in a GenTek plate reader. Thermal ramps (25 to 50 °C) were conducted by heating by 1° with a 3-min equilibration period before taking Abs measurements at 600 nm. The resulting heatmaps were plotted in 3D using a custom Python script.

### Circular dichroism

A solution of 0.05 mg/mL CaHD (1.4 µM) was prepared in buffer (10 mM sodium phosphate pH 7.4, 10 mM NaF). Two hundred microliters of sample were pipetted into a quartz cuvette with a 1-mm pathlength. A temperature ramp of 1 °C/min was used, and the signal at 222 nM was recorded every 0.1°. Three scans were obtained for each of the full-spectrum scans (190 and 260 nM) taken every 5°. Data were collected on a JASCO J1500 spectrometer.

### Proteolysis assay

A 100-µL solution of 1 mg/mL purified protein was coincubated with either 0.12 mg/mL porcine pancreatic elastase (1 µL of 12 mg/mL, Sigma) at room temperature for 0.5 to 24 h, taking time points in between. At each time point, 10 µL of solution was mixed with 10 µL of SDS-loading buffer, and boiled for 5 min to inactivate the protease. Samples were run on a 15% SDS–PAGE gel, and subsequently assayed with mass spectrometry to identify proteolytic fragments.

### Nano differential scanning fluorimetry

Measurements were carried out on a Nanotemper Tycho. Purified protein was inserted into a glass capillary through capillary action and then placed over the instrument mirror. A buffer control was included as a separate capillary. Measurements were conducted between 35 and 95 °C.

### Differential scanning calorimetry

About 1 mg/mL purified protein was dialyzed into 50 mM sodium phosphate, 200 mM NaCl. The sample was heated and cooled between 10 and 75 °C at a heating rate of 0.5 °C/min. Runs with only buffer were conducted and subtracted from the melting curves. Measurements were conducted on a TA Instruments nano DSC in biological duplicate, and data were fit to a two-stage unfolding model.

### Solution NMR measurements

CaHD was triple labeled (^2^H, ^13^C, ^15^N) by growth in M9 minimal media, made using D_2_O (Sigma Aldrich), that contained ^15^N ammonium chloride and uniform ^13^C-glucose (Cambridge Isotopes). Briefly, cells were grown in nonisotopically labeled LB until an OD of 3.0, centrifuged and resuspended in M9-containing labeled glucose and ammonium chloride made up in D_2_O. Cells were further grown to an OD_600_ of 0.6, induced with 1 mM IPTG, and incubated overnight at 20 °C. The protein was then purified using the methods described above for unlabeled protein. Once purified, CaHD was dialyzed into a denaturing buffer (3 M guanidine hydrochloride, 50 mM sodium phosphate, pH 8.0) to exchange backbone deuterium for proteins. CaHD was returned to a nondenaturing buffer by step-wise dialysis into buffers containing 2, 1, 0.5, and finally 0 M guanidine hydrochloride, in steps of 8–12 h each. The sample was then concentrated to a 500-µL at a final concentration of 1 mM and 10% D_2_O. Solution NMR experiments were recorded on a Bruker Avance III NMR spectrometer operating at a ^1^H frequency of 700 MHz. Bruker TopSpin was used for data acquisition with standard 2D and 3D Bruker pulse sequences with water suppression. All spectra were processed with the NMRPipe software package (53) and then visualized and analyzed with the CcpNmr analysis program (54).

## Supplementary Material

pgae139_Supplementary_Data

## Data Availability

Data that support the findings of this study have been deposited at the Small Angle Scattering Biological Data Bank (https://www.sasbdb.org/) and at the Biological Magnetic Resonance Data Bank (https://bmrb.io) at BMRB entry 52238.
