# Supplementary material for: A secreted bacterial protein protects bacteria from cationic antimicrobial peptides by entrapment in phase-separated droplets
Source: PNAS Nexus. 2024 Apr 2;3(4):pgae139. doi: 10.1093/pnasnexus/pgae139 (PMC11022072; doi:10.1093/pnasnexus/pgae139)
Supplement: pgae139_Supplementary_Data [file pgae139_supplementary_data.pdf]

Supplementary Figures

A secreted bacterial protein protects bacteria from cationic antimicrobial peptides by entrapment in phase separated droplets.

Nicholas K. H. Ostan[1], Gregory B. Cole[1] Flora Zhiqi Wang[1,2], Sean E. Reichheld[,3], Gaelen Moore[1,] Chuxi Pan[1], Ronghua Yu[4], Christine Chieh-Lin Lai[1], Simon Sharpe[1,3], Hyun O. Lee[1], Anthony B. Schryvers[4], and Trevor F. Moraes*

There are 7 supplementary figures:

Figure S1: Structure of LbpBs

Figure S2: Affinity capture of CaHD on apo-LF and holo-Lf conjugated sepharose beads.

Figure S3: CaHD alone protects bacteria from CAPs:

Figure S4: Measuring CaHD temperature stability by intrinsic DSF.

Figure S5: Secondary structure of CaHD by solution NMR.

Figure S6: Lactoferrin protease shielding.

Figure S7: Analysis of the LbpB:CAP phase landscape by turbidimetry

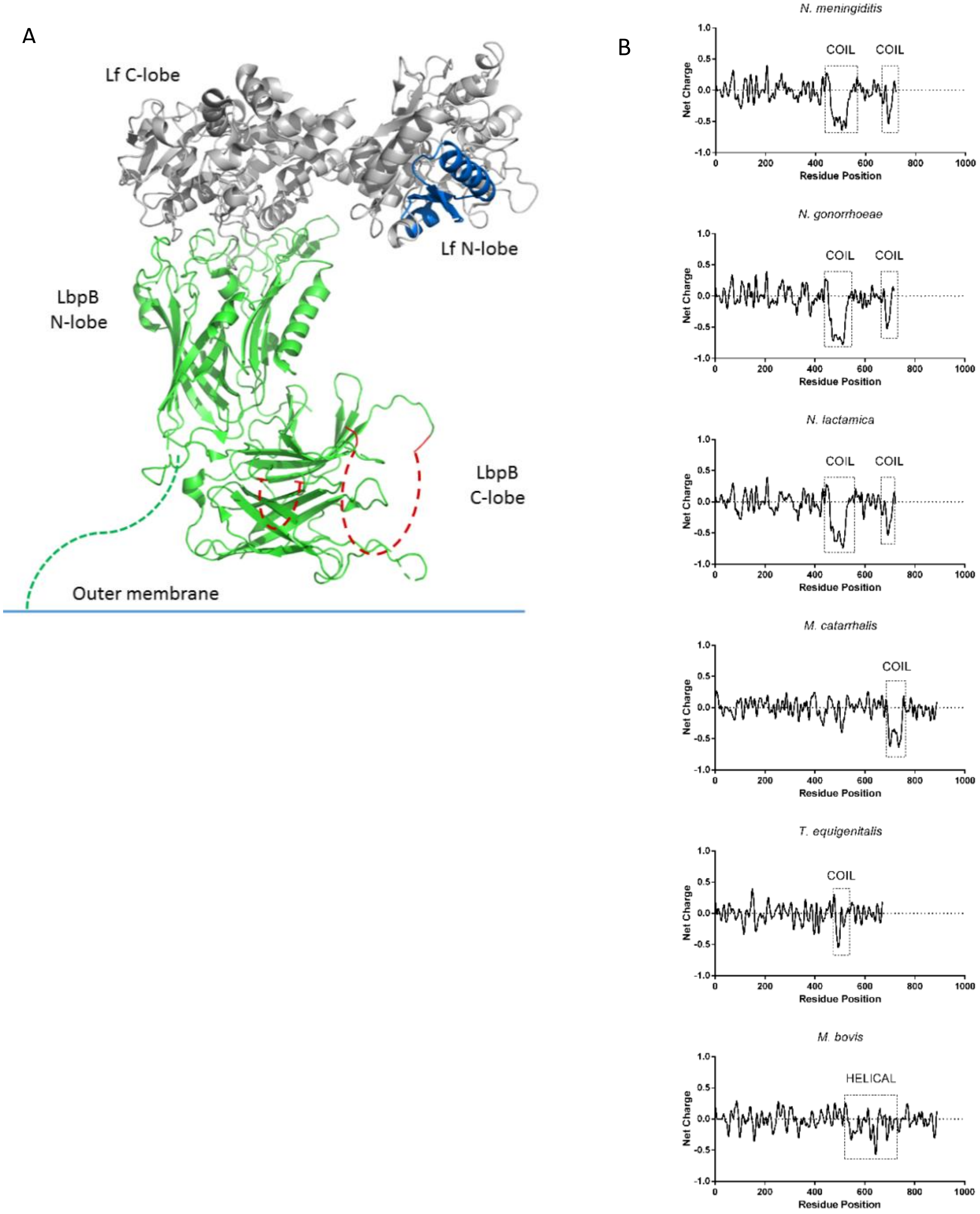

Figure S1: Structure of LbpBs A) PDB 7JRD the crystal structure of N. meningitidis LbpB (in green) complexed with human lactoferrin (in gray). LbpB is comprised of two lobes each made up of a Xstranded beta barrel and a beta handle. Lactoferrin is also a bi-lobed protein comprised of N and C-lobes both of which contain an iron binding site. The N-lobe contains the antimicrobial peptide LfcinB (shown in blue). The N-lobe of LbpB interacts only with the C-lobe of lactoferrin. The LbpB Clobe contains two highly anionic loops not visible in the crystal structure (highlighted in red) **B)** Primary sequence charge and secondary structure analysis. An average charge window of 30 amino acids was used to plot the resultant net charge against its amino acid position. Negative charge clusters are outlined and the predicted secondary structure for the region, as predicted by PSIPRED, is listed above the region.

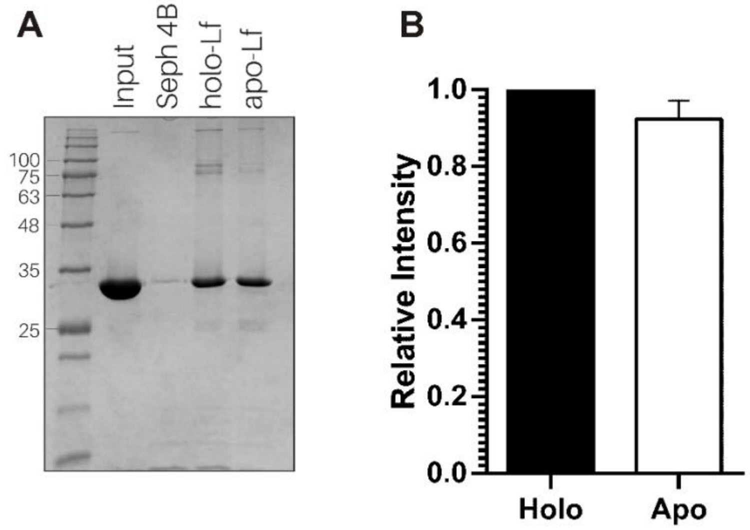

Figure S2: Affinity capture of CaHD on apo-LF and holo-Lf conjugated sepharose beads. A) Representative gel of pull down experiments. **B)** Densitometry quantification from 3 separate gels shown in the adjacent bar graph.

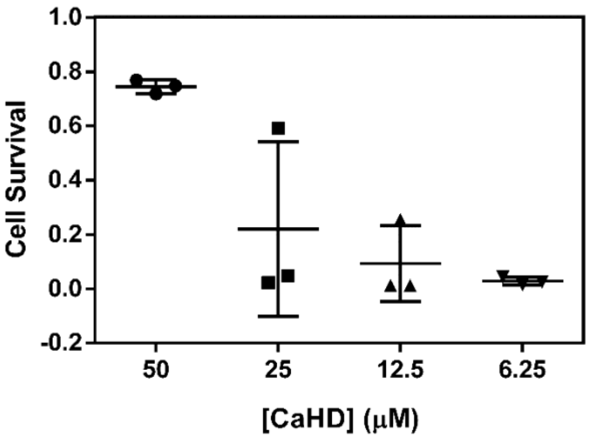

Figure S3: CaHD alone protects bacteria from CAPs: The survival of K12 *E. coli* cells treated with Lf(17-41) at concentrations higher than the MIC (50μM) in the presence of decreasing amounts of CaHD.

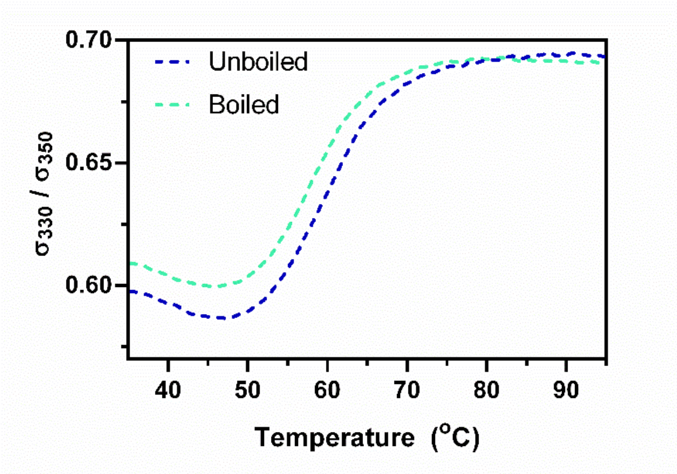

Figure S4: Measuring CaHD temperature stability by intrinsic DSF. Differential scanning calorimetry of CaHD shows a transition occurs at ~57ºC.

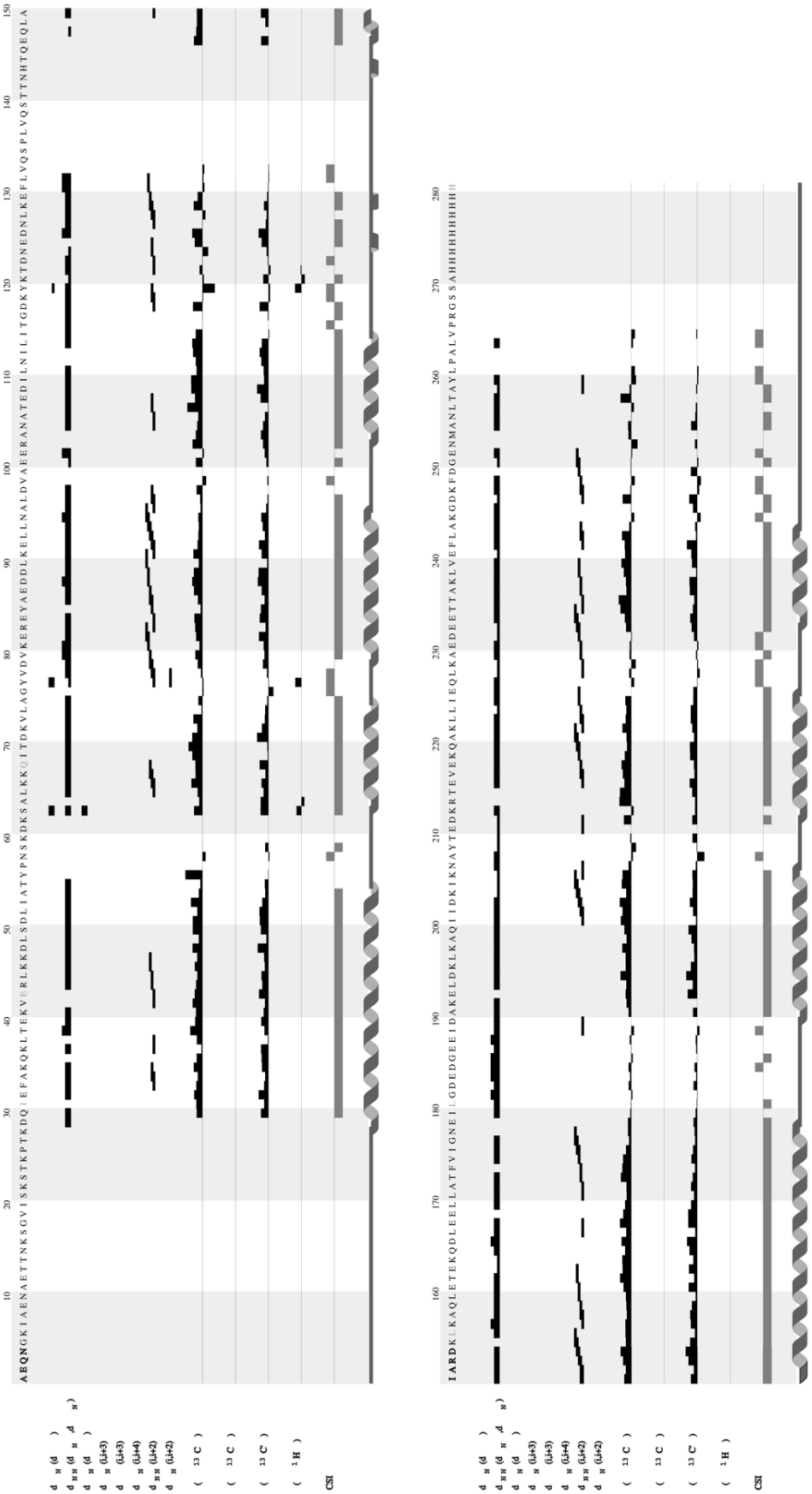

Figure S5: Secondary structure of CaHD by solution NMR. A survey of short and medium range NOEs and analysis of back bone chemical shifts provides a reliable measure of secondary structure.

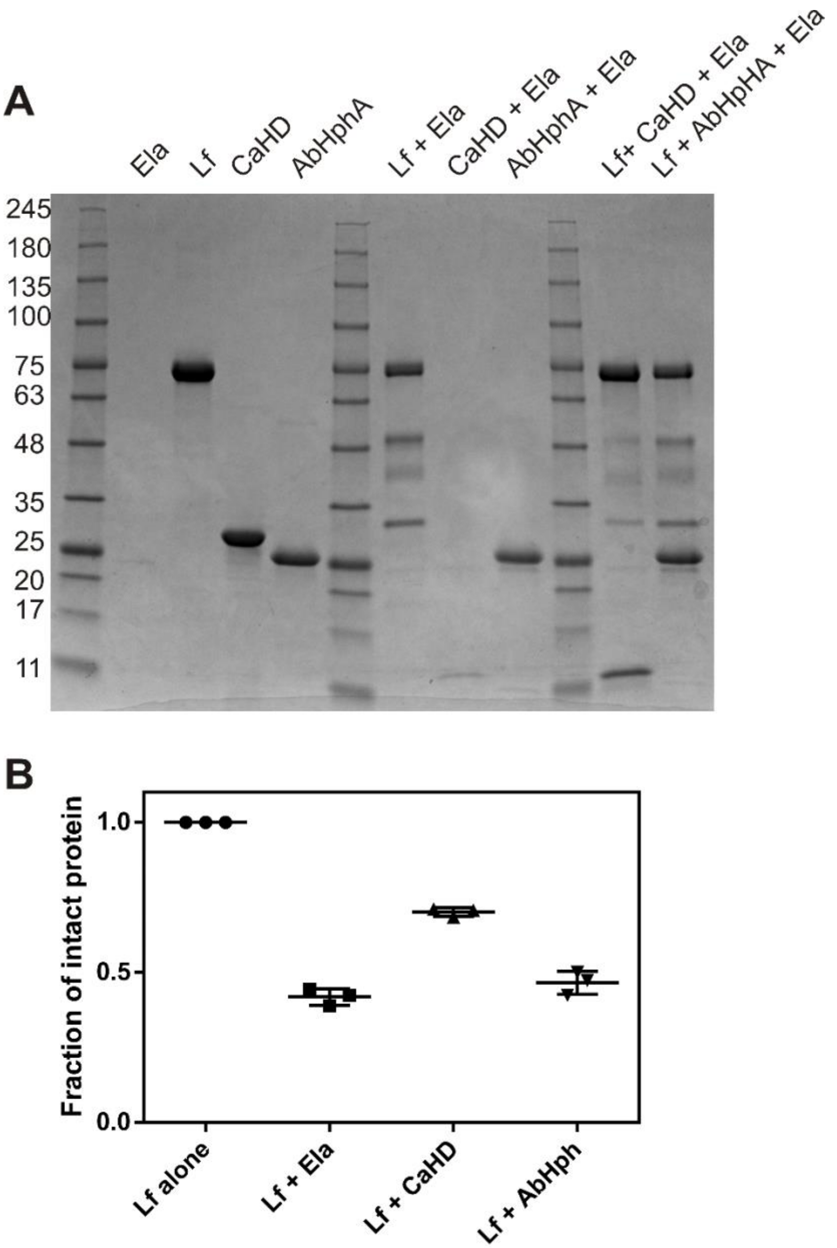

Figure S6: Lactoferrin protease shielding. A) Representative SDS-PAGE showing that when Lf is in the presence of CaHD, it is less prone to proteolysis. Proteins were proteolyzed by porcine elastase (Ela). An unrelated protein of similar size to CaHD, a secreted heme scavenger from *Acinetobacter baumanni*i (AbHpHA), was used as a control to show that the presence of a competing protein is not the driving force of protease resistance. **B)** Quantification of 3 experiments that when Lf is in the presence of CaHD, it is proteolytically processed less than on its own.

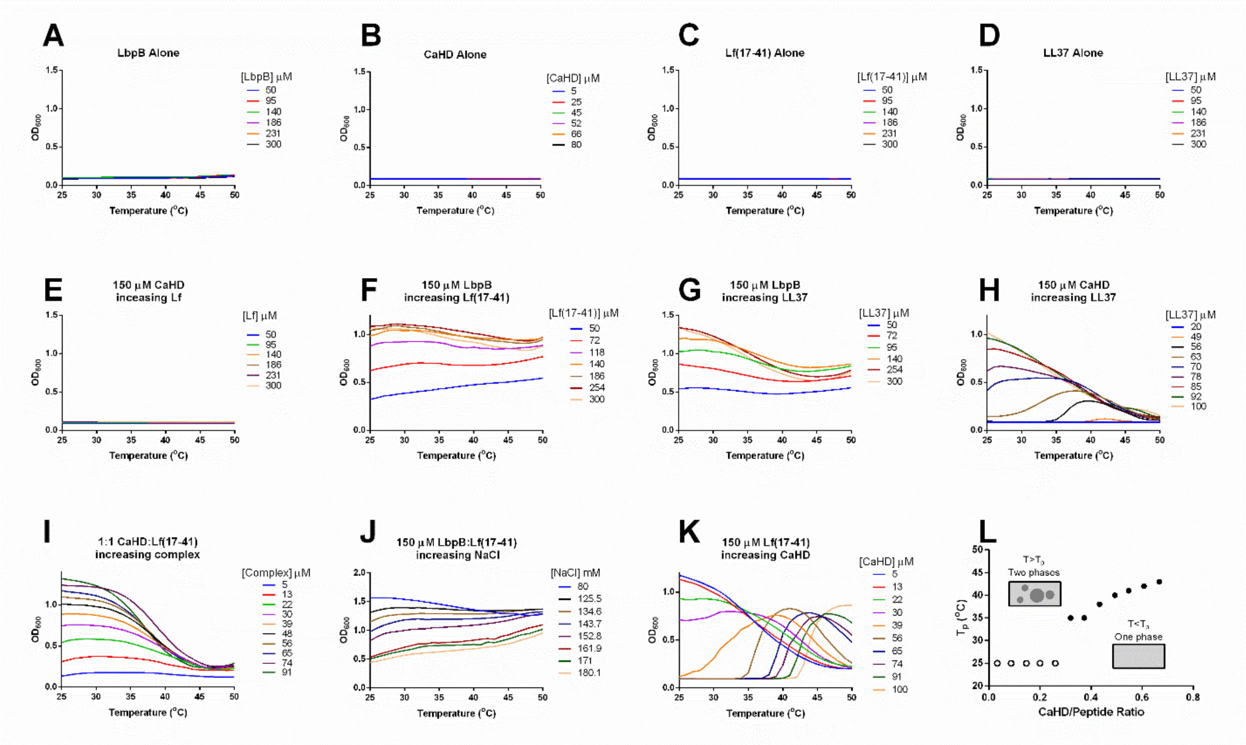

Figure S7: **Analysis of the LbpB:CAP phase landscape by turbidimetry. A-E)** Turbidimetry measurements show that the protein and peptide components used in this study **A)** LbpB, **B)** CaHD, **C)** Lf(17-41) and **D)** LL37 do not phase separate when in isolation nor does the **E)** CaHD:Lf complex. **F-H)** Peptides mixed with CaHD or LbpB result in phase separation and increasing peptide concentration results in increased amounts of phase separation. **I)** Increasing the total amount of CaHD:Lf(17-41) complex increases the amount of phase separation. **J)** The interactions that drive phase separation are disrupted in increasing amounts of salt. **K)** At high peptide to CaHD ratios, there is a higher amount of phase separation. As the peptide to CaHD ratio is lowered the upper critical solution temperature (UCST) shifts to higher temperatures. **L)** The temperature at which phase separation begins (T_p) was computed from, Fig. S7K, and plotted as a function of CaHD:peptide ratio. Open circles indicate ratios where phase separation was already occurring at the lowest temperature measured (25°C) and T_p could not be determined. Higher concentrations of peptide mixed with lower concentrations of CaHD resulted in the highest degrees of phase separation at the lowest temperatures.
